# How Did Urban Land Expand in China between 1992 and 2015? A Multi-Scale Landscape Analysis

**DOI:** 10.1371/journal.pone.0154839

**Published:** 2016-05-04

**Authors:** Min Xu, Chunyang He, Zhifeng Liu, Yinyin Dou

**Affiliations:** 1 Center for Human-Environment System Sustainability (CHESS), State Key Laboratory of Earth Surface Processes and Resource Ecology (ESPRE), Beijing Normal University, Beijing, China; 2 Academy of Disaster Reduction and Emergency Management, Beijing Normal University, Beijing, China; Purdue University, UNITED STATES

## Abstract

Effective and timely quantification of the spatiotemporal pattern of urban expansion in China is important for the assessment of its environmental effects. However, the dynamics of the most recent urban expansions in China since 2012 have not yet been adequately explained due to a lack of current information. In this paper, our objective was to quantify spatiotemporal patterns of urban expansion in China between 1992 and 2015. First, we extracted information on urban expansion in China between 1992 and 2015 by integrating nighttime light data, vegetation index data, and land surface temperature data. Then we analyzed the spatiotemporal patterns of urban expansion at the national and regional scales, as well as at that of urban agglomerations. We found that China experienced a rapid and large-scale process of urban expansion between 1992 and 2015, with urban land increasing from 1.22 × 10^4^ km^2^ to 7.29 × 10^4^ km^2^, increasing in size nearly fivefold and with an average annual growth rate of 8.10%, almost 2.5 times as rapid as the global average. We also found that urban land in China expanded mainly by occupying 3.31 × 10^4^ km^2^ of cropland, which comprised 54.67% of the total area of expanded urban land. Among the three modes of growth—infilling, edge expansion, and leapfrog—edge expansion was the main cause of cropland loss. Cropland loss resulting from edge expansion of urban land totalled 2.51 × 10^4^ km^2^, accounting for over 75% of total cropland loss. We suggest that effective future management with respect to edge expansion of urban land is needed to protect cropland in China.

## Introduction

As the most populous developing country in the world, China has been experiencing rapid and large-scale urban expansion over the last three decades [1–4]. Between 1981 and 2010, China’s urban area increased 4.39-fold, from 7,438 km^2^ to 40,058 km^2^ [5].

China’s widespread urban expansion has resulted in numerous ecological and environmental problems. Rapid urban expansion has resulted in massive cropland loss in China, with mean annual reductions of 1,300 km^2^ between 1990 and 2000 and 2,000 km^2^ between 2000 and 2010 [6,7]. Between 1992 and 2012, 8,647 km^2^ of natural habitat became newly developed urban land, threatening over 100 endangered species, mainly amphibians and reptiles, and resulting in the rapid decline of regional biodiversity [2,8]. China’s urban expansion has influenced the regional climate, with mean surface temperature increases of 0.05°C per decade [9]. In the process of urban expansion, municipal solid waste in China increased at an average annual rate of 9% from 1980 to 2001, polluting soil around landfill sites in a total area of more than 550 km^2^ and about 78% of streams within or around cities [10–12]. In addition, urban areas have become a major source of PM_2.5_, causing severe air pollution and bringing potential health risks to susceptible populations in China [13–15]. To evaluate the ecological and environmental effects of urban expansion in China, timely and accurate elucidation of the process of urban expansion is indispensible.

Several researchers have investigated urban expansion in China at multiple scales. At the national scale, Bai et al. [1] quantified urban expansion and its relationship with economic growth in China from 1990 to 2008. Liu et al. [16] analyzed China’s urban expansion and regional disparities in this expansion from 1990 to 2008. Kuang et al. [17] analyzed the patterns and drivers of urban expansion in China between 1990 and 2010. Wang et al. [18]and Xiao et al. [19] examined China’s urban expansion over the periods 1990–2010 and 1981–2010, respectively. At a regional scale, Li et al. [20] quantified the spatiotemporal patterns of urban expansion in the Yangtze River Delta region from 1979 to 2008. Tian et al. [21] analyzed the growth modes and socioeconomic drivers of urban expansion in Shenzhen from 1973 to 2009. At the city scale, Xu et al. [22], Schneider and Mertes [23], Zhao et al. [24], and Liu et al. [25] analyzed the spatiotemporal patterns of urban expansion of major cities in China before 2010. However, understanding of the most recent dynamics of urban expansion, in the period since 2012, remains inadequate, due to a lack of timely urban land information in China. At present, two types of data are mainly used to understand urban expansion in China: socioeconomic statistical data based on administrative units [26], and medium- to high-resolution remote-sensing data represented by Landsat images [16,17]. However, these two types of data are not appropriate for accurate and timely detection of urban expansion in China at a national scale. Socioeconomic data lack adequate spatial information [27], while medium- to high-resolution remote-sensing data are limited by their spatial coverage and require large amounts of human and computational resources for the extraction of timely data on urban land for the whole nation [16,17].

Nighttime lights provide a novel data source for the detection of China’s urban expansion [28–33]. In 2010, the US National Geophysical Data Center (NGDC) published the fourth version of the time series dataset of the Defense Meteorological Satellite Program (DMSP)—Operational Linescan System (OLS) nighttime stable light (NSL) data [28–31]. In 2013, the NGDC published Version 1 of a dataset of the Suomi National Polar-orbiting Partnership (NPP)—Visible Infrared Imaging Radiometer Suite (VIIRS) nighttime light data [34]. These nighttime light data have appropriate spatial resolution and time series information for the detection of urban expansion at a large scale, providing a reliable data source for understanding urban expansion in China at the national scale [2,35,36]. Some researchers have been working on understanding urban expansion in China using nighttime light data. For example, He et al. [37] examined the process of urbanization in China in the 1990s using nighttime light data. Liu et al. [3] detected urban expansion in China from 1992 to 2008, and He et al. [2] obtained information on urban expansion in China over the period 1992–2012. However, few studies have investigated urban expansion in China since 2012.

In this study, our objective was to quantify the spatiotemporal patterns of urban expansion in China between 1992 and 2015. To achieve this goal, we first extracted information on urban expansion in China between 1992 and 2015 by integrating NSL, vegetation index, and land-surface-temperature data. Then, we analyzed urban expansion and urban growth modes in China at the national and regional scales, as well as at that of urban agglomeration. Finally, we considered the relationship between cropland loss and urban expansion and growth modes in China. Our findings represent the latest investigation on urban expansion in China over the last two decades and will be useful for planning sustainable development in China.

## Materials and Methods

### Data sources

The NPP-VIIRS NSL data for 2015 were obtained from the NGDC website (http://ngdc.noaa.gov/eog/viirs/download_monthly.html, accessed September 30, 2015). These data include monthly average radiance composite images from January to August in 2015 produced using nighttime data from the VIIRS Day/Night Band (DNB) in 15 arc-second geographic grids. These data were preprocessed to remove effects from stray light, lightning, lunar illumination, and clouds [38]. The digital number (DN) value of each pixel represents the radiance of light at night in units of nano-Watts/cm^2^·sr. Following the monthly composition approach, we produced a mean annual value composite image of NPP-VIIRS NSL in 2015.

Moderate-Resolution Imaging Spectroradiometer (MODIS) 8-day composite imagery of nighttime land surface temperature (LST) (MOD11A2-Level 3) was obtained from the data archive and distribution system of the National Aeronautics and Space Administration (NASA) (http://ladsweb.nascom.nasa.gov, accessed September 30, 2015). Each image is composed of average LST values over an 8-day period with a resolution of 1 km [39]. Differences in LST between urban and non-urban areas are much more significant at night than during the day [40,41]. Therefore, MODIS nighttime LST data are better suited for distinguishing between urban and non-urban areas [42]; thus, these data were used in the present study. Following Mildrexler et al. [43], we produced a maximum value composite (MVC) image of LST to capture nighttime LST characteristics in 2015.

The MODIS 16-day composite imagery of the normalized difference vegetation index (NDVI) was also obtained from NASA (http://ladsweb.nascom.nasa.gov, accessed September 30, 2015). Each image was a composite of maximum NDVI values observed over 16 days at 500-m resolution. These data were radiometrically calibrated, precisely georeferenced, and corrected for atmospheric effects before distribution. Furthermore, based on the work of Lu et al. [44] and Cao et al. [45], we produced an MVC image of NDVI for 2015. Although an annual MVC image of NDVI conceals land-cover changes that take place during the year, it can efficiently reduce contamination by clouds while capturing changes in spatial vegetation characteristics [44,45]. All NSL, LST, and NDVI images collected for this study were further reprojected onto the Albers projection and resampled to a spatial resolution of 500 m.

Urban land data in China from 1992 to 2012 were obtained from the urban land dataset developed by He et al. [2]. These data accurately represent urban expansion in China from 1992 to 2012, with spatial resolution of 1 km, average overall accuracy of 95.20%, average quantity disagreement of 2.24%, average allocation disagreement of 2.56%, and an average Kappa value of 0.66.

Five high-quality Landsat images were obtained from the Geospatial Data Cloud, operated by the Chinese Academy of Sciences (http://www.gscloud.cn, accessed September 30, 2015) (Table 1).

**Table 1:** Landsat TM/ETM+ data used in the study.

City covered	Path/row	Data
Beijing	123/32	September 7, 1992; April 17, 2015
Nanjing	120/38	October 20, 1992; June 15, 2015
Kunming	129/43	August 16, 1992; January 5, 2015
Zhengzhou	124/36	October 16, 1992; September 15, 2015
Nanchang	121/40	May 20, 1992; February 14, 2015

The 1990 national land use/cover dataset for China was obtained from the Data Sharing Infrastructure of the Earth System Science at the Chinese Academy of Science (http://www.geodata.cn, accessed September 30, 2015). This dataset was produced at 1-km resolution in the Albers projection through visual interpretation of Landsat Thematic Mapper (TM) images [46]. The proportion of land use/cover for each pixel was extrapolated from the original 30-m pixels. This NLCD map represents actual land-use/cover conditions in China in 1990 fairly accurately [46].

Socioeconomic census data from 1992 and 2013, including the urban population and gross domestic product (GDP) of each province in China, were obtained from the Statistical Database of Economic and Social Development by the National Knowledge Infrastructure of China (http://tongji.cnki.net, accessed September 30, 2015). The administrative boundaries of the provinces were obtained as GIS files from the National Geomatics Center of China.

### Method

#### Acquiring information on urban expansion in China

**Fig 1:**
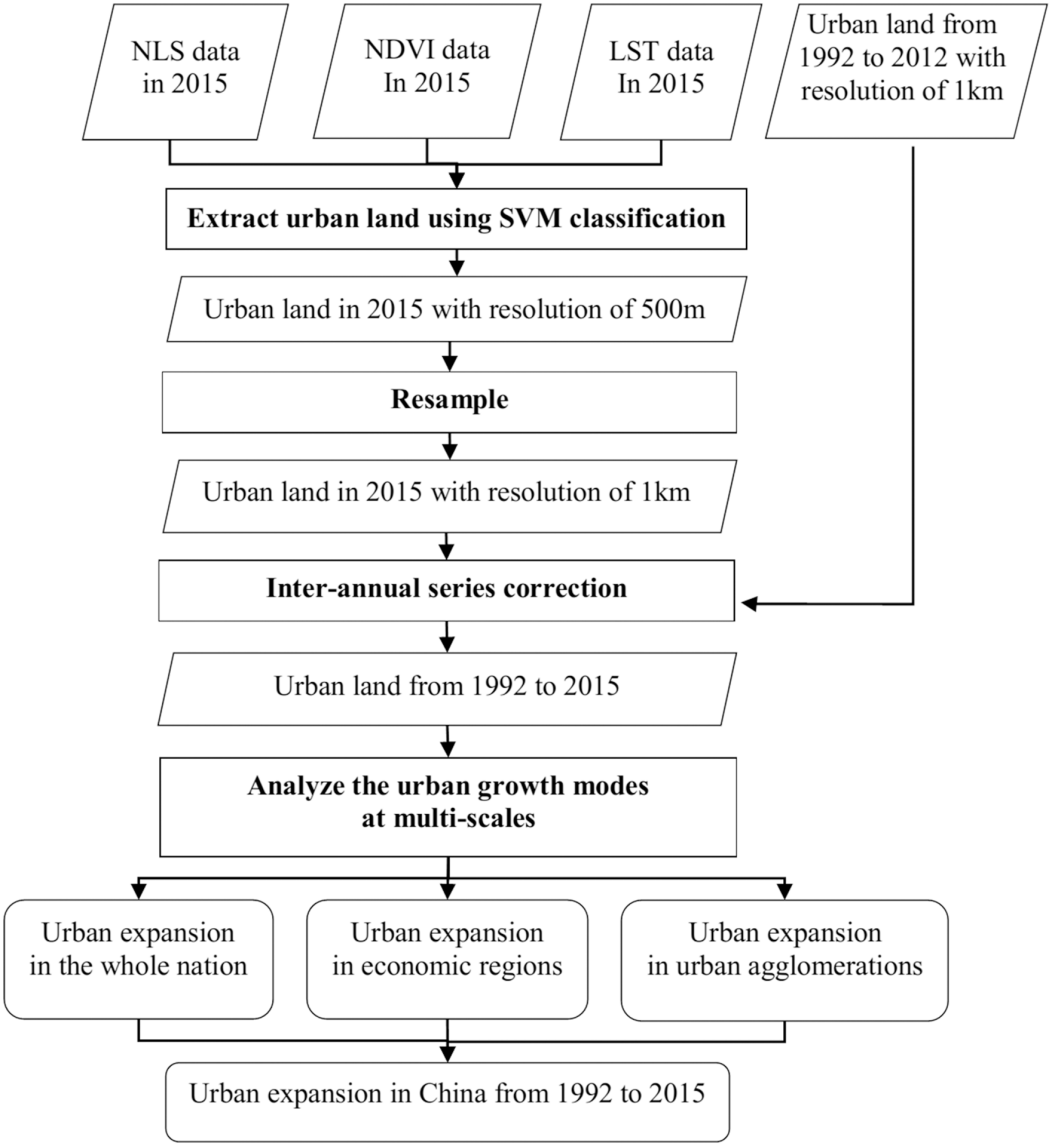
Flow chart of urban expansion analysis process. Note: NSL, nighttime stable light; NDVI, normalized difference vegetation index; LST, land surface temperature; SVM, Support Vector Machine.

Accurate acquisition of time series information on urban expansion in China between 1992 and 2015 (Fig 1) is the foundation of our research. Following the methods suggested by Yang et al. [47] and He et al. [2], we extracted urban land areas of China in 2015, using the stratified support vector machine (SVM) method based on NSL, NDVI, and LST data. The SVM method, proposed by Vapnik in 1995, is one of the non-parametric learning methods based on statistical learning theory [48]. It can effectively solve the learning problems resulting from few training samples, nonlinearity, and high-dimensional data and, thus, it has been used widely to extract urban land from multiple remote-sensing data [45, 48]. First, China was divided into eight economic regions that are relatively homogeneous within themselves and distinct from each other: Northeast China (NEC), Northern Coastal China (NCC), Southern Coastal China (SCC), Eastern Coastal China (ECC), the Middle Reaches of the Yellow River (MRYLR), the Middle Reaches of the Yangtze River (MRYTR), Southwest China (SWC), and Northwest China (NWC) [49]. Second, sample NSL, LST, and NDVI values were taken across the various socioeconomic and physical environments within each economic region. SVM classification was subsequently performed using adaptive post-classification to create a binary map of urban land for China in 2015 at 500-m resolution, based on the original NPP-VIIRS NSL, NDVI, and LST images. To maintain consistency with the urban land data from 1992 to 2012 extracted by He et al. [2], the urban land image in 2015 was resampled to a spatial resolution of 1 km, based on the majority rule. In addition, with reference to Liu et al. [27] and He et al. [2], urban areas in China were assumed to have grown continuously outward between 1992 and 2015, and a pixel with a spatial resolution of 1 km, detected as urban by nighttime light data in a previous year remained urban in later years. Using this assumption, urban land in 2015 was inter-annual series corrected with urban land from 1992 to 2012. Data processing can be summarized using the following logical function:

$$DN_{\left(2015,i\right)}^{R}=\{\begin{array}{ll} 1 & DN_{\left(2012,i\right)}^{}=1\\ DN_{\left(2015,i\right)}^{O} & otherwise \end{array}$$ (1)

Where $DN_{\left(2015,i\right)}^{R}$ is class value (urban or non-urban) at the *i*th pixel in 2015 after the inter-annual series correction, $DN_{\left(2015,i\right)}^{O}$ is class value at the *i*th pixel in 2015 before the inter-annual series correction. $DN_{\left(2012,i\right)}^{}$ is the class value at the *i*th pixel in the 2012 urban land image obtained from He et al. [2]. A class value of 1 represents urban, while 0 represents non-urban. By performing the inter-annual series based on this formula, we obtained the urban land from 1992 to 2015 in China with a spatial resolution of 1km.

#### Analyzing urban expansion and urban growth modes in China

Using the landscape expansion index (LEI) proposed by Liu et al. [50], we analyzed the modes of urban expansion and urban growth in China at the national and regional scales, as well as that of the urban agglomeration, between 1992 and 2015. The LEI of each new urban patch was calculated year by year using the following formula:

$$LEI=\frac{A_{0}}{A_{0}+A_{v}}\times 100$$ (2)

where *A*_*0*_ is the intersection between the buffer around a new urban patch and the previously existing urban land, and *A*_*v*_ is the intersection between the buffer zone and the previously non-urban area. Based on the LEI, urban growth can be classified into three modes: infilling, edge expansion, and leapfrog [50]. The infilling mode of urban growth refers to gaps between old urban patches being filled with new urban patches (i.e., *LEI* is between 50 and 100). The edge-expansion mode of urban growth is when a new urban patch expands from the edges of an existing urban patch (i.e., *LEI* is between 0 and 50). The leapfrog mode of urban growth is when a new urban patch is isolated from the old ones (i.e., *LEI* is equal to 0) [50].

## Results

### Accuracy assessment

**Fig 2:**
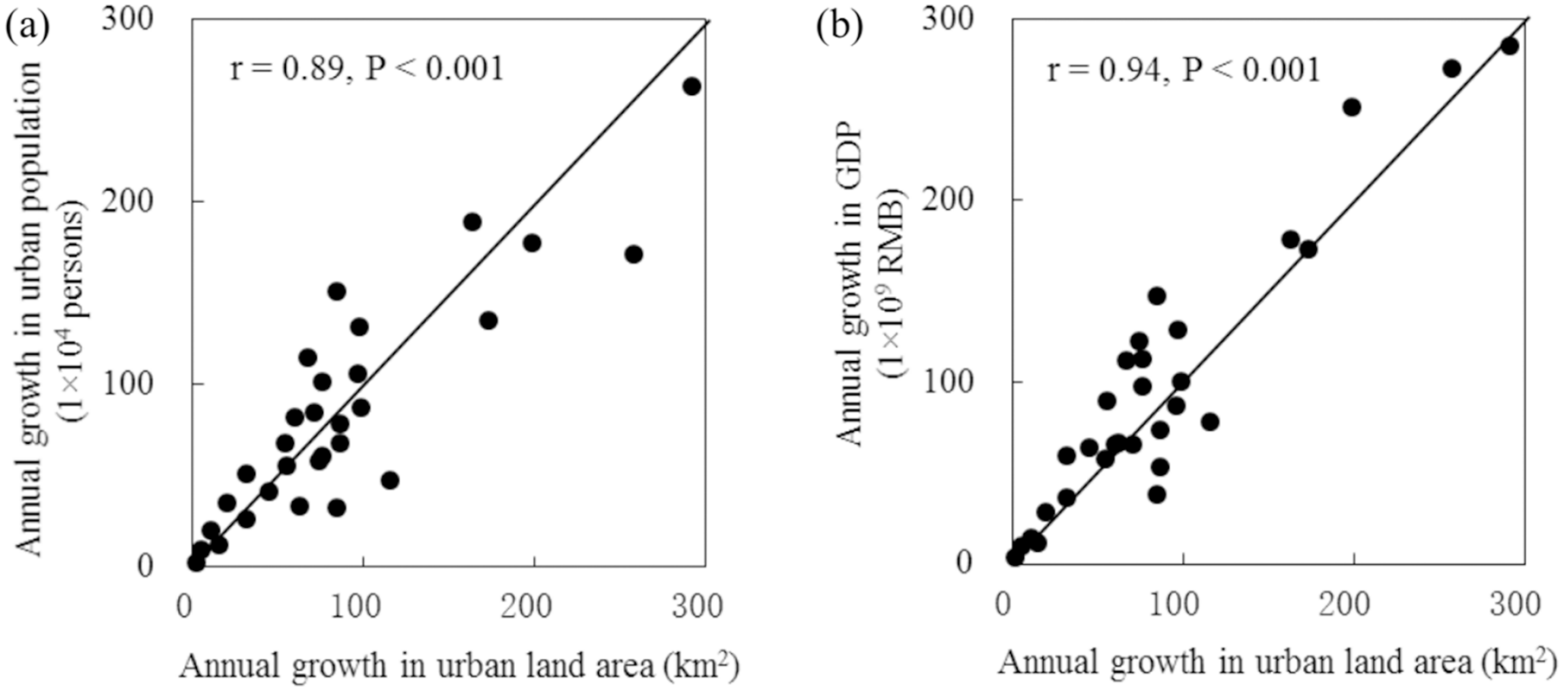
Correlation between annual growth in urban land area and annual growth in urban population and gross domestic product (GDP) between 1992 and 2015. (a) Correlation between urban land area and urban population; (b) Correlation between urban land area and GDP.

**Fig 3:**
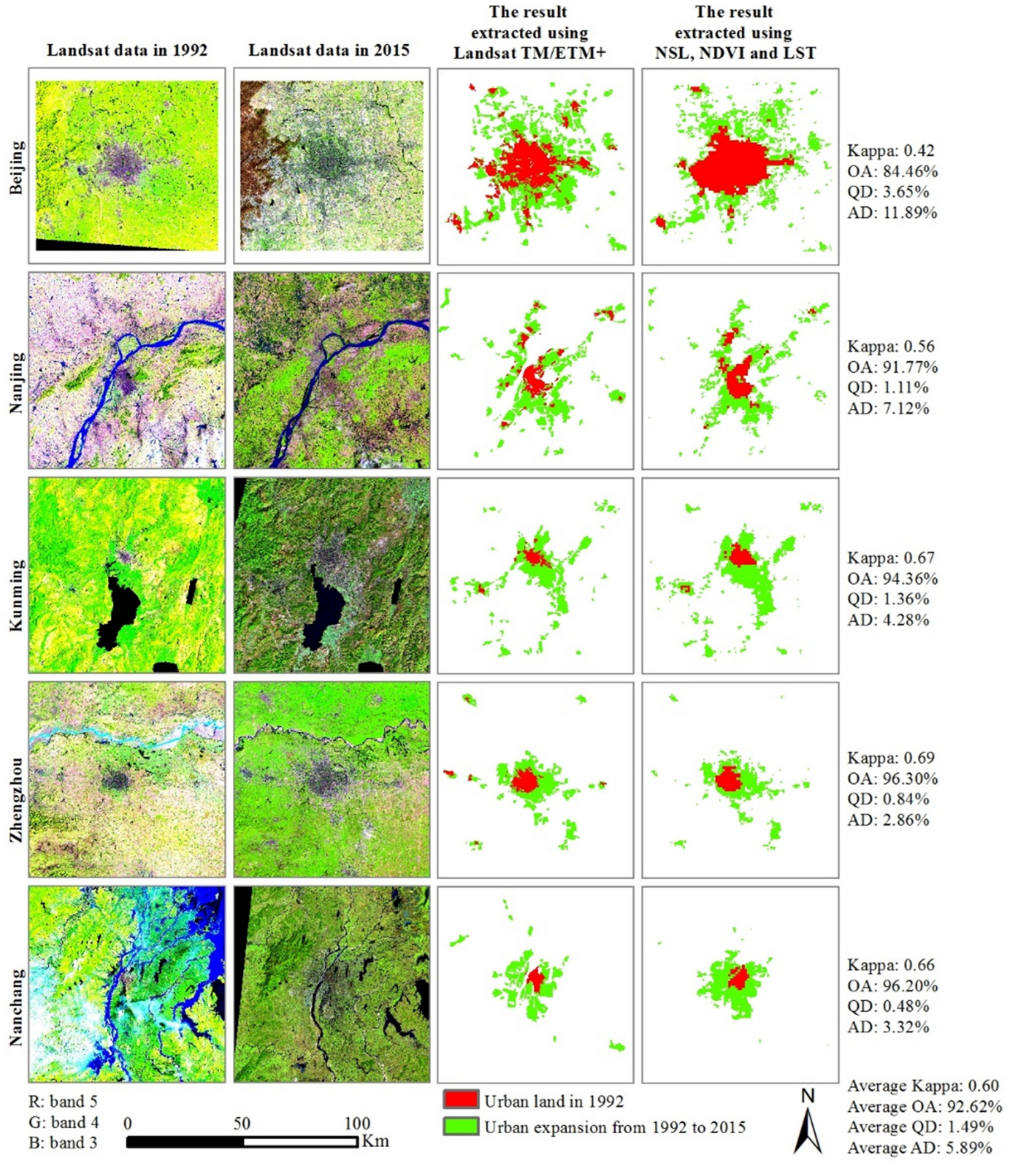
Accuracy assessment of urban expansion between 1992 and 2015.

Following Liu et al. [27] and He et al. [2], we used socioeconomic census data and medium- to high-resolution remote sensing data to assess the accuracy of the estimated urban expansion in China between 1992 and 2015. First, the relationships between the mean annual growth of urban land area, the mean annual growth of the urban population, and GDP were analyzed at the provincial level. The assessment showed that our estimated results had a strong correlation (R > 0.8) with census data for urban population growth and GDP at a significance level of 0.001 (Fig 2). In addition, we assessed the spatial accuracy of our estimated urban expansion with reference to urban land extracted from Landsat TM/ETM+ images. In this assessment process, five important capital cities with various levels of urbanization were selected as sample areas. The assessments resulted in an average Kappa value of 0.60, an average overall accuracy (OA) of 92.62%, an average quantity of disagreement (QD) of 1.49%, and an average allocation of disagreement (AD) of 5.89% (Fig 3). The relatively high accuracy suggests that urban expansion across China between 1992 and 2015 was well captured in our study.

### Urban expansion at the national scale

**Fig 4:**
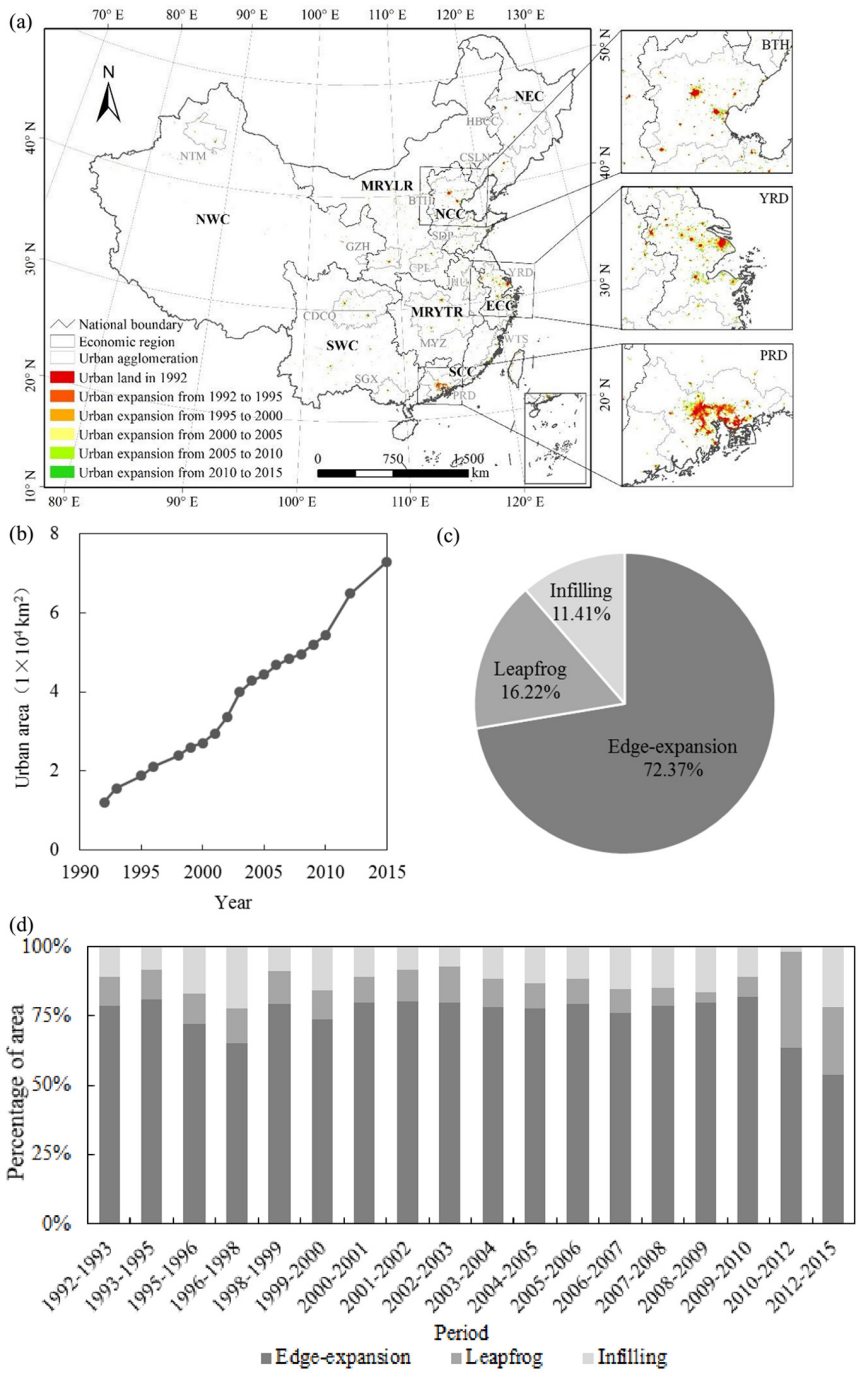
Urban expansion in China between 1992 and 2015. (a) Spatial patterns of urban land; (b) Change in urban land area; (c) Percentage of expanded urban land area in different urban growth modes; (d) Change in urban growth modes. Notes: Economic regions include Eastern Coastal China (ECC), Middle Reaches of the Yellow River (MRYLR), Middle Reaches of the Yangtze River (MRYTR), Northern Coastal China (NCC), Northeast China (NEC), Northwest China (NWC), Southern Coastal China (SCC), and Southwest China (SWC). Urban agglomerations include Harbin-Changchun (HBCC), Central and Southern Liaoning (CSLN), Beijing-Tianjin-Hebei (BTH), Shandong Peninsula (SDP), Guanzhong (GZH), Central plains (CPL), Jianghuai (JHU), Yangtze River Delta (YRD), Chengdu-Chongqing (CDCQ), Middle Yangtze (MYZ), Western Taiwan Straits (WTS), Southern Guangxi (SGX), Pearl River Delta (PRD), and Northern Tianshan Mountains (NTM).

China experienced rapid urban expansion from 1992 to 2015 (Fig 4). Urban land increased from 1.22× 10^4^ km^2^ in 1992 to 7.29 × 10^4^ km^2^ in 2015, with an average annual growth rate of 8.10%, while the global average between 1990 and 2000 was only 3.20%, according to Angel et al. [51]. The annual growth rate of urban land in China was thus almost 2.5 times as rapid as the global average. Specifically, in the period of 1992–2000, urban land increased from 1.22 × 10^4^ km^2^ to 2.71 × 10^4^ km^2^, with annual growth of 1,868 km^2^ or 10.54%. In the period of 2000–2010, urban land increased from 2.71 × 10^4^ km^2^ to 5.42 × 10^4^ km^2^, with annual growth of 2,713 km^2^ or 7.19%. In the period of 2010–2015, urban land increased from 5.42 × 10^4^ km^2^ to 7.29 × 10^4^ km^2^, with annual growth of 3,728 km^2^ or 6.09% (Fig 4b).

Meanwhile, edge expansion was the primary mode of urban expansion in China during the period 1992–2015 (Fig 4c). Expansion of urban land by this mode was 4.39 × 10^4^ km^2^, accounting for 72.37% of the total area of expanded urban land. Expanded urban land areas resulting from the leapfrog and infilling modes were 9,848 km^2^ and 6,925 km^2^, accounting for 16.22% and 11.41% of the total expanded urban land, respectively. The percentage of urban land resulting from edge expansion was dramatically larger than the values for the other two modes (Fig 4c, Table 2). Specifically, the annual expansion of urban land by edge expansion was 1,410 km^2^ between 1992 and 2000, 2,156 km^2^ between 2000 and 2010, and 2,219 km^2^ between 2010 and 2015, accounting for 75.70%, 79.48%, and 59.53% of the total expanded urban land, respectively (Fig 4d).

**Table 2:** Urban expansion in China between 1992 and 2015.

Economic region*	Growth in urban land			Growth in urban land resulting from different growth modes					
	Area (km^2^)	Percentage**	Annual growth rate	Edge-expansion		Leapfrog		Infilling	
				Area (km^2^)	Percentage***	Area (km^2^)	Percentage***	Area (km^2^)	Percentage***
ECC	11,658	19.20%	9.76%	8,833	75.77%	1,083	9.29%	1,742	14.94%
SCC	10,353	17.05%	6.27%	7,368	71.17%	1,162	11.22%	1,823	17.61%
NCC	9,509	15.66%	7.07%	7,291	76.67%	1,181	12.42%	1,037	10.91%
SWC	8,103	13.35%	11.96%	5,830	71.95%	1,645	20.30%	628	7.75%
MRYLR	7,846	12.92%	8.28%	5,285	67.36%	2,110	26.89%	451	5.75%
MRYTR	6,885	11.34%	9.92%	5,175	75.16%	1,055	15.32%	655	9.51%
NEC	3,457	5.69%	5.73%	2,559	74.09%	478	13.84%	420	12.16%
NWC	2,903	4.78%	10.24%	1,600	55.12%	1,134	39.06%	169	5.82%
Total	60,714	100.00%	8.10%	43,941	72.37%	9,848	16.22%	6,925	11.41%

*Please refer to Fig 4 for names of economic regions.

**Expansion of urban land in region as a percentage of total expansion of urban land area in China.

***Expansion of urban land as a percentage of total expansion of urban land area in the corresponding economic region.

### Urban expansion at the regional scale

**Fig 5:**
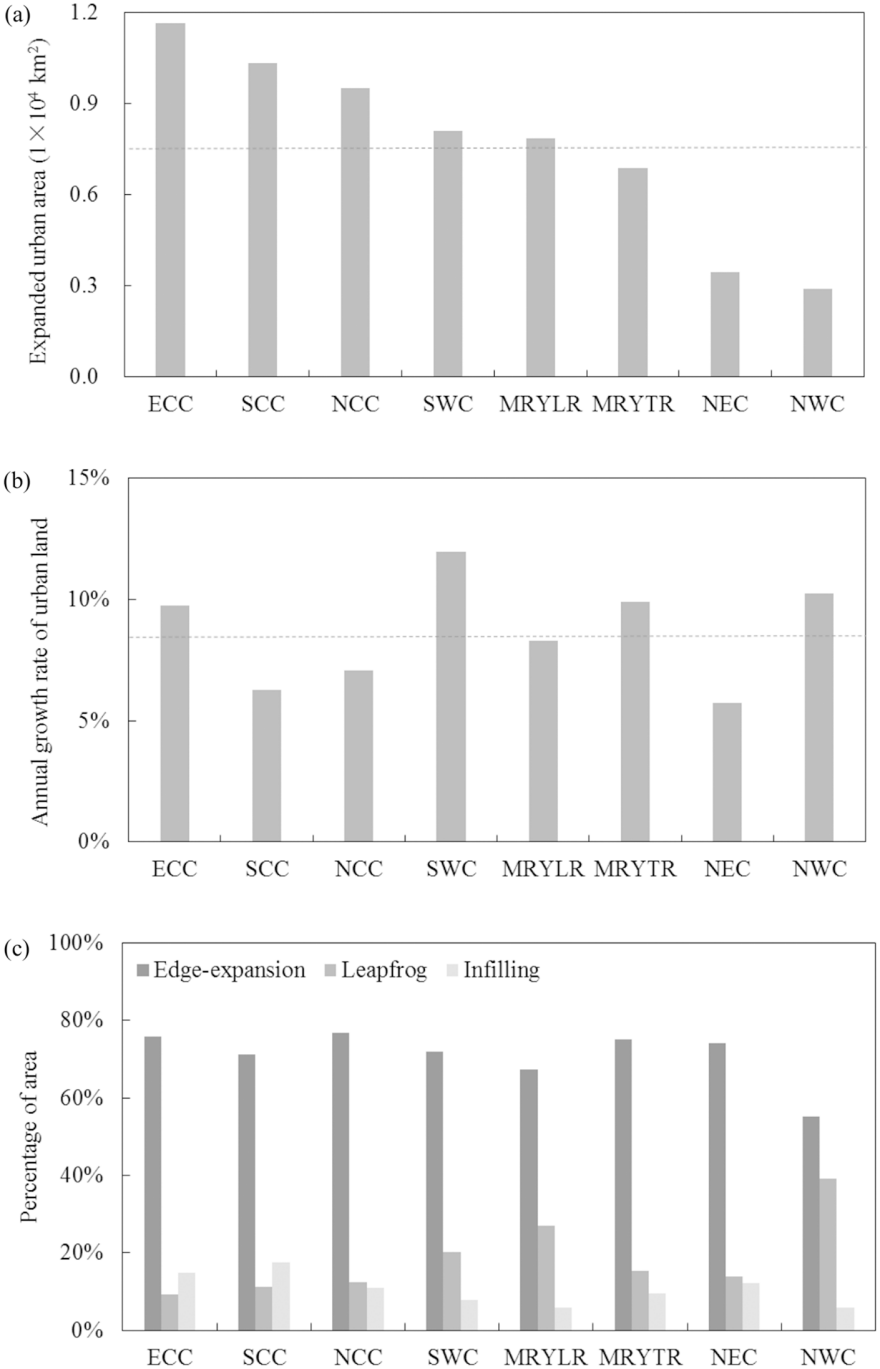
Urban expansion between 1992 and 2015 across economic regions. (a) Expanded urban land area; (b) Annual growth rate of urban land; (c) Percentage of expanded urban land area in different urban growth modes. Dotted line represents regional average. Note: Please refer to Fig 4 for names of economic regions.

Across the eight economic regions, the average urban land area increased from 1,519 km^2^ in 1992 to 9,108 km^2^ in 2015, a net growth of 7,589 km^2^, or an average annual growth rate of 8.65%. The average area of urban land resulting from edge expansion was 5,493 km^2^, accounting for 70.90% of the total area of expansion of urban land (Fig 5).

East Coastal China (ECC) experienced much greater urban expansion than did other economic regions. Between 1992 and 2015, the urban land area in ECC increased from 1,551 km^2^ to 1.32 × 10^4^ km^2^, a net growth of 1.17 × 10^4^ km^2^, or 1.54 times the average of eight economic regions. In addition, the average annual growth rate of urban land in ECC was 9.76%, or 1.13 times the average level across economic regions. Edge expansion was the dominant urban growth mode in ECC from 1992 to 2015. The area of urban land resulting from edge expansion was 8,833 km^2^, accounting for 75.77% of the total area of expanded urban land in ECC, which was nearly 5% higher than the average.

Northwest China (NWC) had the smallest area of urban expansion. Between 1992 and 2015, urban land increased from 345 km^2^ to 3,248 km^2^, with an average annual growth rate of 10.24%. The net growth of urban land in NWC was 2,903 km^2^, which was about one-third of the economic regional average. Edge expansion was the dominant urban growth mode in NWC, amounting to 1,600 km^2^ and accounting for 55.12% of the total expansion of urban land.

### Urban expansion at the urban agglomeration scale

**Fig 6:**
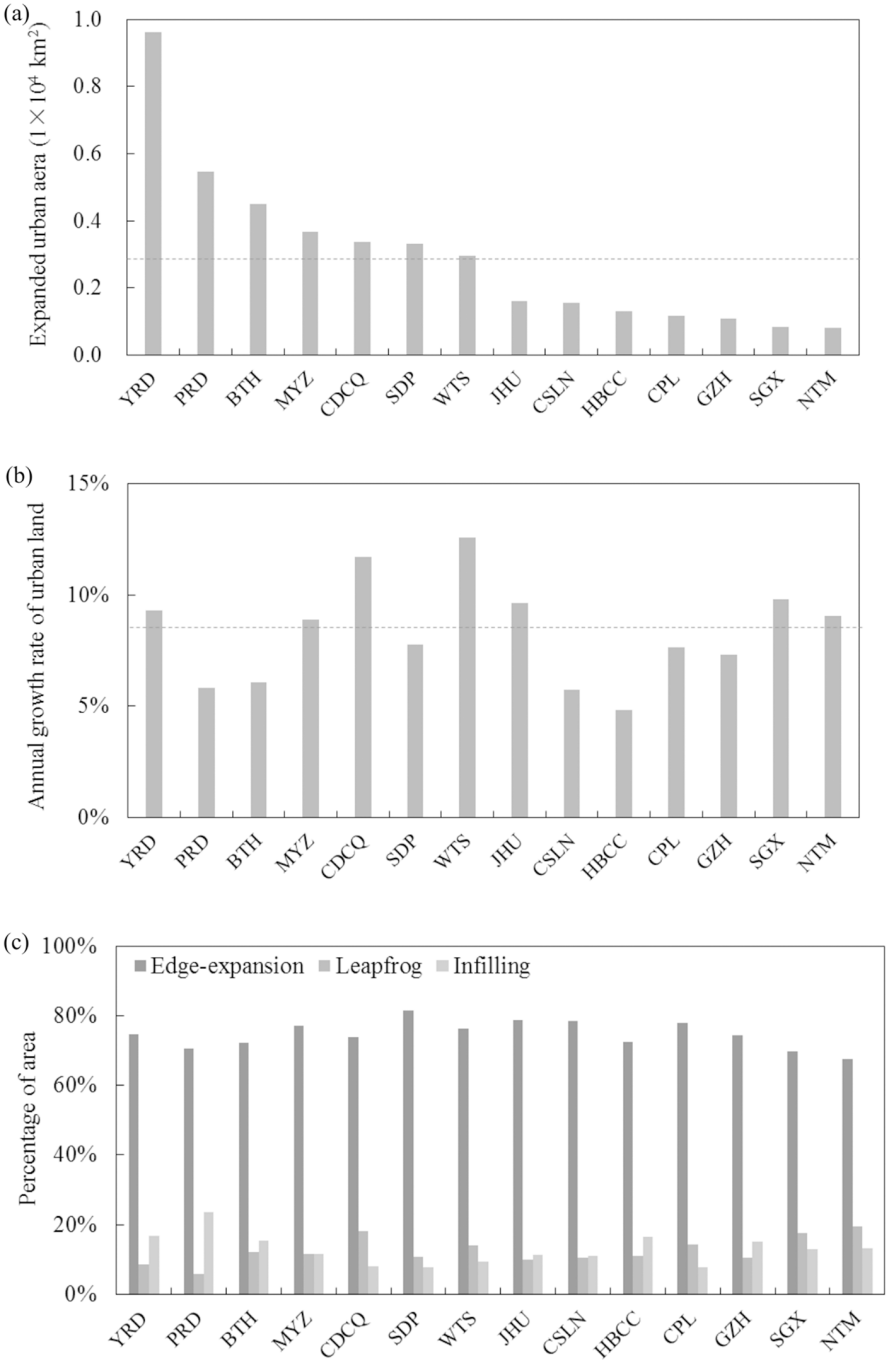
Urban expansion between 1992 and 2015 across urban agglomerations. (a) Expanded urban land area; (b) Annual growth rate of urban land; (c) Percentage of expanded urban land area under different urban growth modes. Dotted line represents the regional average. Note: Please refer to Fig 4 for corresponding urban agglomeration names.

In the 14 main urban agglomerations planned by the Chinese government in 2014 (The CPC Central Committee and The State Council, 2014; Fang, 2015), urban land increased from 9,066 km^2^ in 1992 to 5.03 × 10^4^ km^2^ in 2015, an annual growth rate of 7.73%. The area of new urban land was 4.12 × 10^4^ km^2^, accounting for 67.87% of the total expansion of urban land area at the national level. The area of urban land resulting from edge expansion was 3.08 × 10^4^ km^2^, or 74.77% of the total expansion of urban land in these urban agglomerations (Fig 6, Table 3).

**Table 3:** Urban expansion across urban agglomerations between 1992 and 2015.

Urban agglomeration*	Growth in urban land			Growth in urban land resulting from different growth modes					
	Area (km^2^)	Percentage**	Annual growth rate	Edge-expansion		Leapfrog		Infilling	
				Area (km^2^)	Percentage***	Area (km^2^)	Percentage***	Area (km^2^)	Percentage***
YRD	9,625	15.85%	9.30%	7,183	74.63%	827	8.59%	1,615	16.78%
PRD	5,456	8.99%	5.84%	3,848	70.53%	321	5.88%	1,287	23.59%
BTH	4,504	7.42%	6.08%	3,260	72.38%	547	12.14%	697	15.48%
MYZ	3,679	6.06%	8.90%	2,835	77.06%	421	11.44%	423	11.50%
CDCQ	3,353	5.52%	11.69%	2,480	73.96%	604	18.01%	269	8.02%
SDP	3,303	5.44%	7.78%	2,691	81.47%	354	10.72%	258	7.81%
WTS	2,965	4.88%	12.58%	2,266	76.42%	418	14.10%	281	9.48%
JHU	1,591	2.62%	9.64%	1,254	78.82%	159	9.99%	178	11.19%
CSLN	1,540	2.54%	5.74%	1,208	78.44%	161	10.45%	171	11.10%
HBCC	1,302	2.14%	4.83%	943	72.43%	143	10.98%	216	16.59%
CPL	1,168	1.92%	7.63%	911	78.00%	167	14.30%	90	7.71%
GZH	1,074	1.77%	7.33%	800	74.49%	112	10.43%	162	15.08%
SGX	841	1.39%	9.79%	586	69.68%	147	17.48%	108	12.84%
NTM	801	1.32%	9.06%	541	67.54%	155	19.35%	105	13.11%
Total	41,202	67.87%	7.73%	30,806	74.77%	4,536	11.01%	5,860	14.22%

*Please refer to Fig 4 for names of urban agglomerations.

**Expansion of urban land in region as a percentage of total expansion of urban land area in China.

***Expansion of urban land as a percentage of total expansion of urban land area in the corresponding region of urban agglomeration.

Among all urban agglomerations, the greatest urban expansion was that of the Yangtze River Delta (YRD). Between 1992 and 2015, the urban land area increased from 1,429 km^2^ to 11,054 km^2^ in YRD, a net growth of 9,625 km^2^, or 3.27 times the average of all the urban agglomerations. The average annual growth rate of urban land in YRD was 9.76%, or 1.20 times the average value for all urban agglomerations. Edge expansion was the primary mode of urban expansion in YRD, accounting for 7,183km^2^ and representing 74.63% of the total expansion of urban land.

The smallest area of urban expansion was for urban agglomeration in the Northern Tianshan Mountains (NTM). Between 1992 and 2015, the urban land area increased from 126 km^2^ to 927 km^2^, an average annual growth rate of 9.06%. The net growth of urban land in the NTM was 801 km^2^, about one quarter of the average. The dominant urban growth mode was edge expansion, which accounted for 541 km^2^ or 67.54% of the total expansion of urban land.

## Discussion

### Urban land in China expanded mainly by occupying cropland

**Fig 7:**
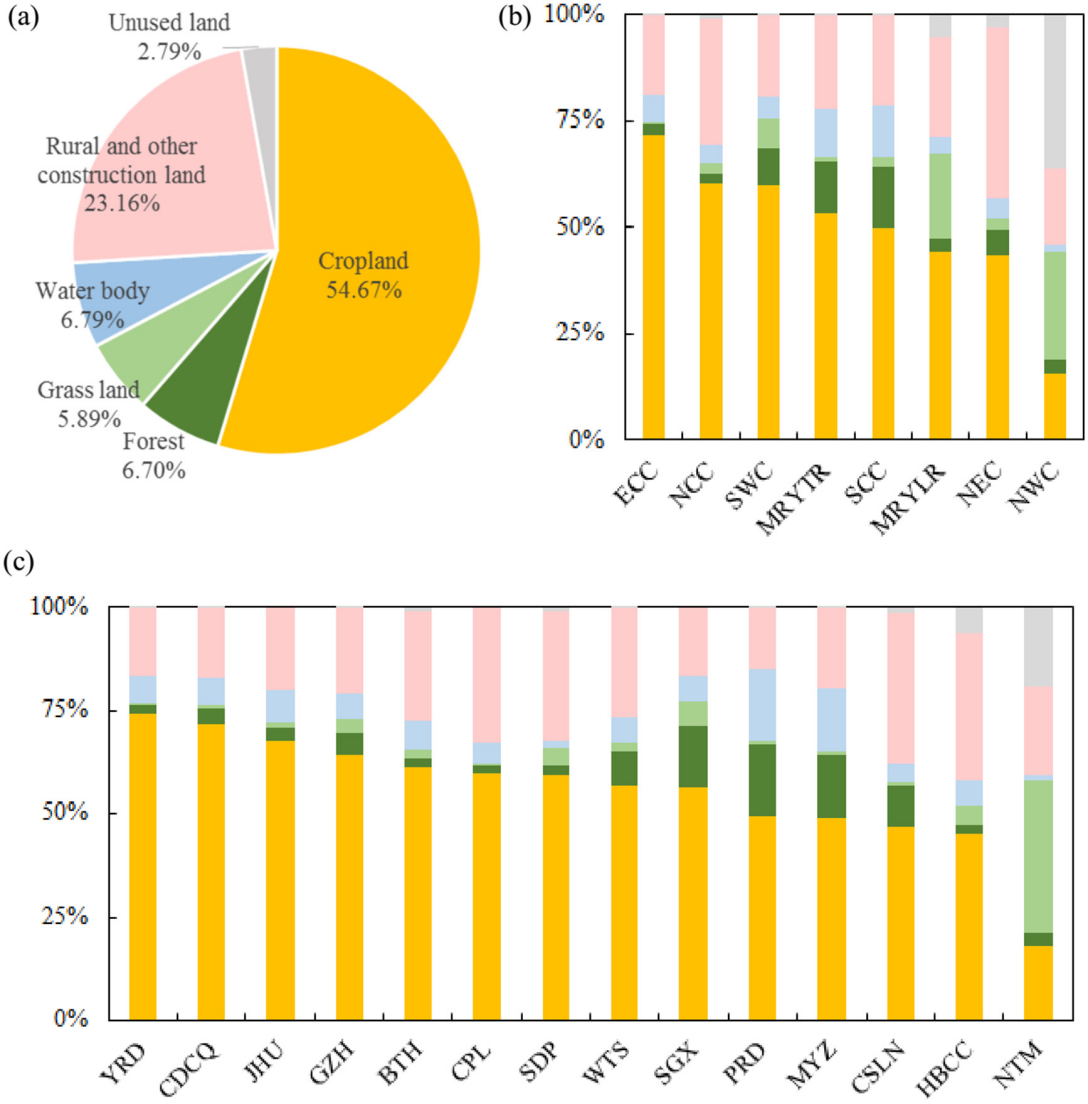
Occupied land during urban expansion between 1992 and 2015. (a) In China; (b) By economic region; (c) By urban agglomeration. Note: Please refer to Fig 4 for names of economic regions and urban agglomerations.

Urban expansion in China caused a massive cropland loss of 3.31 × 10^4^ km^2^, accounting for 54.67% of the total area of urban expansion between 1992 and 2015 (Fig 7a). The area of rural and other construction land transformed to urban land was 1.40 × 10^4^ km^2^, or 23.16% of the total expansion in urban land (Fig 7a). Areas of forest, grassland, and bodies of water were reduced by approximately 4,000 km^2^, or about 6% of the total urban expansion area (Fig 7a). Less than 2,000 km^2^ of unused land were converted to urban land, accounting for nearly 3% of the total area of urban expansion (Fig 7a). The area of cropland occupied by urban land was much greater than that of other land types (Fig 7a).

Five economic regions experienced severe cropland loss: ECC, NCC, SWC, MRYTR, and SCC. In each of these five economic regions, the area of cropland converted to urban land amounted to over 3,500 km^2^, accounting for more than 50% of the total area of urban expansion (Fig 7b). Among these regions, ECC experienced the greatest cropland loss (Fig 7b). As a result of urban expansion in ECC between 1992 and 2015, cropland decreased by 8,324 km^2^, accounting for 71.44% of total urban expansion in this region, which was 21.67% higher than the average among the eight economic regions over the same period.

In 14 urban agglomerations, 2.48 × 10^4^ km^2^ of cropland in total were converted to urban land between 1992 and 2015, accounting for 55.74% of the total area of urban expansion. It is worth noting that YRD experienced the most severe cropland loss; about 7,158 km^2^ of cropland was converted to urban land, accounting for 74.39% of new urban land, which was nearly 20% higher than the average (Fig 7c).

### Lost cropland in China was mainly occupied by edge expansion of urban land

**Fig 8:**
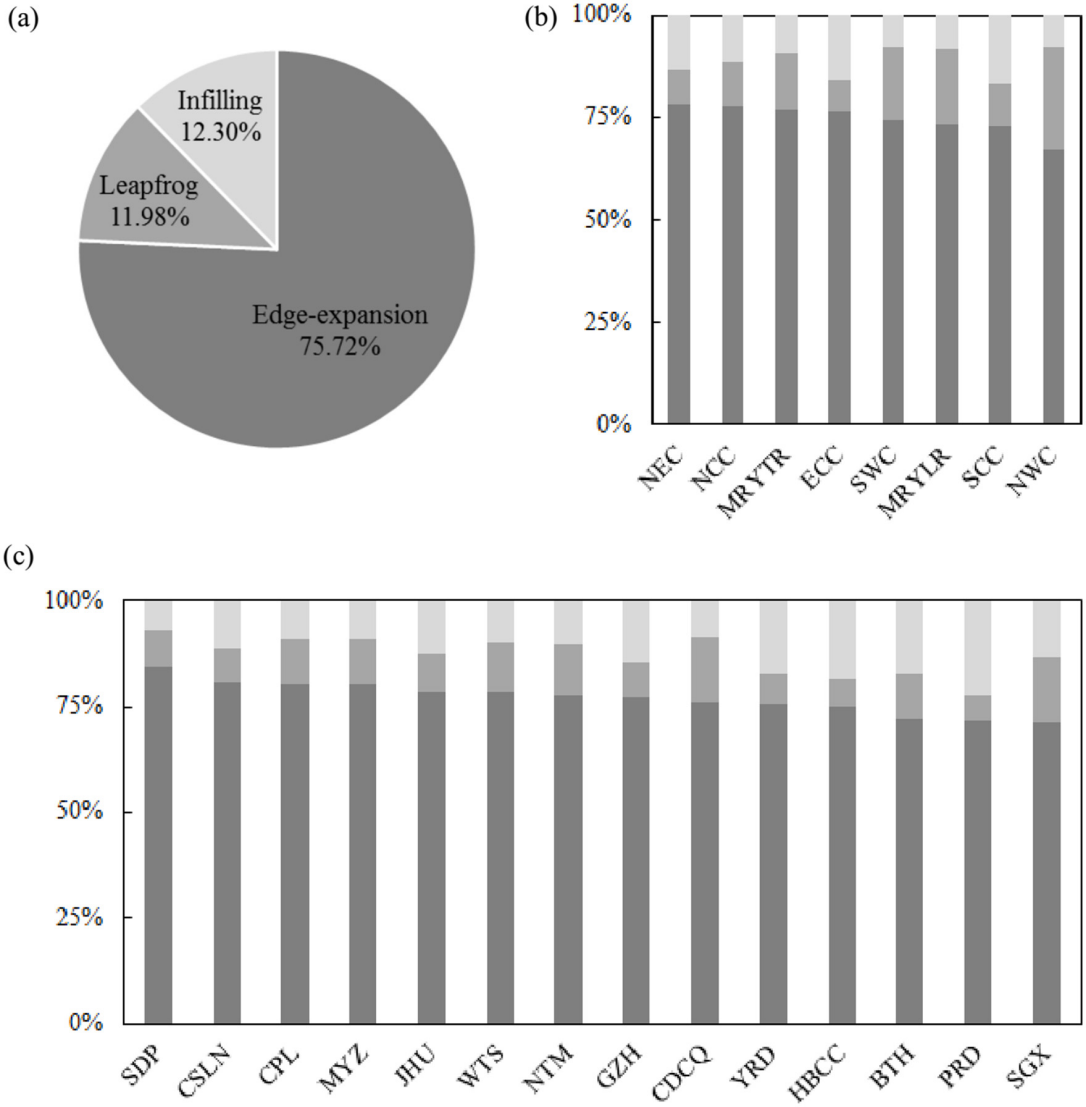
Cropland loss caused by urban expansion in different urban growth modes. (a) In China; (b) By economic region; (c) By urban agglomeration. Note: Please refer to Fig 4 for names of economic regions and urban agglomerations.

Between 1992 and 2015, the cropland loss resulting from edge expansion of urban land was 2.51 × 10^4^ km^2^, accounting for 75.72% of total cropland loss during the process of urban expansion in China (Fig 8a). In the eight economic regions, the percentage of cropland lost to edge expansion of urban land all surpassed 65% (Fig 8b). Among the regions, NEC experienced the most severe cropland loss from edge expansion of urban land, cropland occupied by expansion of urban edges amounted to 1,176 km^2^, which was nearly 80% of the total cropland loss (Fig 8b). In 14 urban agglomerations, cropland decreased by 1.90 × 10^4^ km^2^ due to edge expansion of urban land, accounting for 76% of total cropland converted to urban land. In each urban agglomeration, the percentage of cropland lost to edge expansion of urban land surpassed 70% (Fig 8c). In particular, the urban agglomeration in the Shandong Peninsula (SDP) lost 1,656 km^2^ of cropland due to the edge expansion of urban land, or nearly 85% of the total area of cropland lost to urban expansion (Fig 8c).

Our findings are consistent with the results of previous studies. According to Liu et al.[46], urban expansion is the main cause of cropland loss. Between 1990 and 2000, 45.96% of the total decrease in cropland area resulted from conversion to urban land, and this increased to 55.44% in the period 2000–2010 [7]. In addition, these authors found that the average annual loss of cropland resulting from urban expansion increased from 13 million hectares in the 1990s to 20 million hectares in the 2000s, indicating an increasingly severe conflict between urban expansion and cropland conservation.

Since 2014, the Chinese government has been promoting the National New-type Urbanization Plan to develop human-oriented, efficient, and sustainable cities [52]. China’s urbanization level is expected to reach 60% by 2020 under this plan [53], implying that urban land area will continuously increase [54]. In consideration of this situation, we suggest that effective management of edge expansion of urban land is needed to protect cropland in China.

### Limitations and future perspectives

There are some limitations to our research. For example, after extracting urban land data for China in 2015 –by integrating NPP-VIIRS NSL data, NDVI data, and LST data—we further obtained urban land data for China for 1992 to 2015, with inter-annual series correction based on the assumption that a pixel detected as urban in an earlier year would remain urban in later years [27]. Such inter-annual correction may have exaggerated the extent of urban land in China in 2015. In addition, information on urban expansion is limited by the resolution of nighttime light data, which are acquired only at 1-km resolution, making it difficult to analyze urban growth modes with higher precision [50]. Particularly, the urban land away from city centers with their area less than 1 km^2^ usually cannot be recognized by using our approach, resulting in underestimation of the urban expansion area with the leapfrog mode to some extent.

However, urban expansion dynamics in China can be revealed at a finer spatial resolution of 500 m with the support of the NPP-VIIRS nighttime light data [55]. Additionally, the impact of urban expansion on the quality of cropland can be evaluated using approaches based on cropland productivity, such as the methodology of Agro-Ecological Zones (AEZ) developed by the Food and Agriculture Organization of the United Nations (FAO) [56,57]. Furthermore, we will quantitatively investigate the impact of urban expansion using models such as the Integrated Valuation of Ecosystem Services and Tradeoffs (InVEST) and the model of Weather Research and Forecasting (WRF) [58,59].

## Conclusion

China experienced rapid and massive urban expansion between 1992 and 2015. Urban land increased nearly fivefold, from 1.22 × 10^4^ km^2^ in 1992 to 7.29 × 10^4^ km^2^ in 2015, with an average annual growth rate of 8.10%, almost 2.5 times as rapid as the global average. At a regional scale, ECC accounted for the largest urban expansion at 1.17 × 10^4^ km^2^, nearly one fifth of the total expansion of urban areas in China. The 14 main urban agglomerations planned by the Chinese government are hotspots of urban expansion, where the new urban land area reached 4.12 × 10^4^ km^2^, accounting for nearly two-thirds of the national total. The urban agglomerations of the YRD experienced the most massive urban expansion, of 9,625 km^2^, or one sixth of the national total.

Urban land in China expanded mainly by occupying cropland, resulting in a massive cropland loss of 3.31 × 10^4^ km^2^ and accounting for 54.67% of the total expansion of urban land. The greatest cropland loss occurred in ECC, where 8,324 km^2^ or 71.44% of the expansion of urban land was converted from cropland. The urban agglomeration of the YRD experienced the most severe cropland loss, of 7,158 km^2^ or 74.39% of the area of urban expansion.

Edge expansion of urban land was the main cause of cropland loss in China. Urban land resulting from edge expansion amounted to 4.39 × 10^4^ km^2^, or over two thirds of the total area of expanded urban land during the period 1992–2015. Cropland loss resulting from edge expansion of urban land was 2.51 × 10^4^ km^2^, accounting for over 75% of the total cropland loss. The percentage of total cropland loss due to edge expansion of urban land was nearly 80% in NEC, reaching almost 85% in the urban agglomeration of SDP. More attention should be given to the edge expansion of urban land to protect cropland in the context of further urbanization of China.
